# How Workaholic Leadership Affects Employee Self-Presentation: The Role of Workplace Anxiety and Segmentation Supplies

**DOI:** 10.3389/fpsyg.2022.889270

**Published:** 2022-05-12

**Authors:** Qi Zeng, Xin Liu

**Affiliations:** School of Public Administration and Policy, Renmin University of China, Beijing, China

**Keywords:** workaholic leadership, employee self-presentation, workplace anxiety, segmentation supplies, conservation of resources theory

## Abstract

In an increasingly competitive and performance-oriented society, workaholic leadership is becoming increasingly common and is even embraced and supported by many organizations. However, previous studies have not paid sufficient attention to the impact of workaholic leadership on employee psychology and behavior. This study, based on the conservation of resources (COR) theory, explores the effect of workaholic leadership on employee self-presentation. Through an empirical analysis of 256 employees’ questionnaires, we found a significant positive impact between workaholic leaders and employee self-presentation. This process was achieved through the partly mediating mechanisms of employee workplace anxiety. Concurrently, segmentation supplies negatively moderated the relationship between workplace anxiety and self-presentation and the overall mediating mechanism. These findings provide important insights into the underlying mechanisms of workaholic leadership and employee behavior, which can be utilized to improve employee wellbeing and provide positive organizational outcomes.

## Introduction

Today, it is not rare to hear someone describe their leader as a workaholic. Leaders with this trait devote themselves to their jobs with intensity and put in longer hours than their organizations officially require at the expense of personal time that could be devoted to family responsibilities. With the increasing intensity of external competition, both organizations and employees are facing growing pressure. To this end, it is typical to see long working hours and frequent overtime work in all walks of life (Weng and Xi, 2011). Although some employees will likely devote more time and energy than others to their work to gain a competitive advantage, leaders may more likely need to spend more physical and mental resources on challenging tasks and their leadership responsibilities. Moreover, increasing competition requires superiors to exert more effort in controlling the whole team (Yan and Yang, 2010). Therefore, workaholic leadership has gradually become an increasingly common leadership style in modern Chinese organizations. Previous studies also support the view that workaholism is more prevalent among managers than in others (Andreassen et al., 2012; Taris et al., 2012). Concurrently, organizations highly praise workaholic leadership to a certain extent (McMillan and Northern, 1995).

Workaholic leadership has also attracted considerable academic attention. For instance, previous studies have defined two core characteristics of workaholism: overwork and intrinsic drive (Weng and Zang, 2016). Overwork refers to leaders who work long hours, often work harder than the job demands, and exceed organizational expectations. Intrinsic drive reflects the leader’s obsession with work; this is displayed in their behavior. For example, they still think about work even when leaving the job. Scholars refer to leaders with workaholic traits as workaholic leaders (Clark et al., 2016; Pan, 2018; She et al., 2020). Our study employs the definition used in previous studies and describes workaholic leadership as characterized by a leader’s addiction to work, manifesting as an inner drive to work excessively (Clark et al., 2016; She et al., 2021). Moreover, previous studies have found that workaholic leadership affects team creativity (Li et al., 2021), subordinate performance (She et al., 2020), and employee withdrawal (Pan, 2018). However, there is a lack of research examining workaholic leadership, especially how workaholic leadership affects employees’ psychology and behavior. Since leaders have many work resources, will employees “dance to their leader’s pipe?” Owing to the high power-distance in Chinese contexts (Lin et al., 2013), some employees may maintain a good relationship with their superiors by engaging in ingratiating behavior (i.e., pretending to work hard) (Higgins et al., 2003; Liu et al., 2015). This strategy effectively influences, controls, and manipulates the superior’s impression (Liden and Mitchell, 1988; Conroy and Motl, 2003). As a typical form of ingratiation (Jones, 1967; Tedeschi and Melburg, 1984), self-presentation creates the desired impression (Leary and Downs, 1995) for gaining social acceptance (Leary, 2001). People may self-present to fulfill the expectation of others in the workplace (Baumeister, 1982; Leary and Kowalski, 1990), which varies according to the situation (e.g., pretending to be busy and concentrate at work) (Schlenker and Leary, 1982). Therefore, in the Chinese context, does workaholic leadership motivate employees to display ingratiating behavior like self-presentation, leading to trying their utmost to show their busyness and seriousness to avoid appearing out of tune with workaholic leadership? This behavior should be further explored.

The conservation of resources (COR) theory explains this phenomenon from a research perspective. Individuals will try their best to obtain and protect their physical and mental resources from depletion (Hobfoll, 1989). As the loss of physical and mental resources causes tension and stress in cognition, individuals may take defensive measures to avoid further loss of resources (Hobfoll et al., 2018). Based on this theory, when workaholic leadership encourages the whole team to work long hours and with high intensity, the employees’ physical and mental resources are likely to be depleted, and they are likely unable to obtain timely replenishment. To control the loss of physical and mental resources and mitigate the psychological loss caused by workplace anxiety, employees may seek the resources from the leader, and thus, turn to ingratiation (Hobfoll, 2001; Liu et al., 2015). Consequently, work stress and negative emotions are likely to lead to workplace anxiety. Therefore, workaholic leadership may mediate employee self-presentation through workplace anxiety.

In addition, workaholic leaders consciously cram their own personal and family time and their employees’ time. Suppose such workaholism is not only a personal leadership style but has also become the mainstream work atmosphere supported or advocated for by the entire organization. In that case, employees will be more pressured by work. Moreover, the loss of physical and mental resources will be more serious, leading employees to self-presentation as a coping strategy. That is, if superiors adopt a workaholic style, while the organization itself tacitly approves or even calls for work encroaching on employees’ private time, employees exhibit more pronounced self-presentation. In contrast, if the superior adopts a workaholic style, but the organization advocates for boundaries between work and life, so that employee physical and mental resources can be restored, the employee might not rely on self-presentation. Segmentation supplies is used for describing the degree to which an organization supports employees not to do their jobs in their private time (Kreiner, 2006). Therefore, employees can avoid the continuous loss of physical and mental resources caused by workaholic leaders and reduce self-presentation in an organization with high segmentation supplies. Similarly, employees may ultimately engage in more visible self-presentation behavior when segmentation supplies is low. Last but not least, there should be noticed that, as a form of ingratiation, the purpose of self-presentation could be divided into resources loss perspective (biased to COR theory) and resources acquisition perspective (biased to impression management theory), with the former involving resources that tend to be conditional (e.g., promotions), while the latter favoring energetic resources (e.g., physical, and mental aspects). Our study focuses on the loss of energetic resources (e.g., workplace anxiety) caused by workaholic leaders via the perspective of COR theory to verify the effect of self-presentation on alleviating resource depletion by empirical study.

This study attempts to uncover the mechanism and process of the influence of workaholic leadership on employee self-presentation from the perspective of COR theory and to clarify the mechanism of “workaholic leadership → workplace anxiety → employee self-presentation.” In practice, this study is expected to help organizations dialectically understand the possible influence of workaholic leaders on employees. This enhanced understanding will help reduce blind praise for workaholic leaders and avoid embarrassing situations where employees suffer anxiety and engage in self-presentation, which is not beneficial to the organization. Thus, a literature summary is presented below, and hypotheses based on this are presented.

## Literature Review and Research Hypothesis

### The Effect of Workaholic Leadership on Employee Self-Presentation

Similarity attraction theory (Byrne, 1971) and social generalization theory (Brewer, 1979) point out that individuals with a higher similarity will attract each other, actively form close-knit groups, and will consciously or unconsciously exclude employees with lower similarity. Leaders have the power and resources to determine salary increases and promotions for their subordinates. To avoid being criticized by leaders for not being active at work, employees tend to adopt behaviors and strategies similar to leaders and are willing to show their enthusiasm for work to gain recognition from leaders and become their “insiders.” In short, an employee will adopt strategies, such as self-presentation as an ingratiatory strategy, to increase the leader’s perception of their similarity and create the illusion of being busy at work.

Self-presentation is a typical ingratiation strategy (Jones, 1967; Tedeschi and Melburg, 1984). Individuals sacrifice more for work or engage in work beyond their designated tasks so that the audience thinks they are contributing a lot to their job (Jones and Pittman, 1982). They may present themselves in the ways they desire to be seen by others (Goffman, 1959) or in the ways that fulfill the expectation of others (Baumeister, 1982; Leary and Kowalski, 1990). That is, individuals strive to appear busy, dedicated, and tireless at work, similar to workaholic leaders’ characteristics, to enhance their superiors’ goodwill and evaluation. Therefore, employees with workaholic leaders may cater to leaders’ attitudes toward work to gain the leader’s recognition and favor. They will do so by either remaining enthusiastic about their work or appearing busy to mislead the leader. However, workaholic leaders often work overtime and even deprive their subordinates of private time to complete more work (Friedman and Lobel, 2003). As previous studies indicate, a workaholic leader would drain employees’ mental resources and cause negative results like emotional exhaustion and a decline in wellbeing (Clark et al., 2016; Kim et al., 2020). In particular, emotionally drained individuals may avoid or withdraw as coping strategies (Leiter, 1991). According to the COR theory (Hobfoll et al., 2018), individuals would strive to maintain and protect their resources from this threat. Self-presentation is an effective way to recoup or protect resources form further loss by pretending to be hard-working. Thus, subordinates may pretend to work hard instead of being genuinely devoted to working. This behavior can not only mislead leaders and give them the illusion that subordinates are working hard (to avoid being criticized, isolated, or excluded by leaders) but can also prevent physical and mental resources from being overconsumed.

In addition, there are three explanations for employees’ motivation to self-present themselves. First, even if employees initially choose to work hard, workaholic leaders are likely to take a toll on their subordinates’ physical and mental health (Fassel, 1990) and wellbeing (Clark et al., 2016) in the long run. Due to the continuous loss of energy resources, it is difficult for employees to maintain enthusiasm for their work over long periods. A workaholic leader may occupy their subordinates’ private time with more work. As a result, employees may become exhausted and ultimately have to engage in self-presentation to protect remaining resources without being criticized or labeled as slacking. Second, from a fairness perspective (Adams, 1965), when their colleagues easily obtain the leader’s recognition by self-presentation, it signals that everyone should ingratiate the leader by self-presenting instead of working hard. Ingratiation (including self-presentation) does gain positive reviews and resources (Zhang and Jia, 2016). Third, employees who prefer to self-present may be composed of a group. They may exclude those who do not employ the same work strategy due to the fear of being exposed. Some employees also turn to self-presentation strategies to avoid workplace rejection (Zhang et al., 2018).

Based on the above, we propose the following hypothesis:

**H1:** Workaholic leadership exerts a positive effect on employee self-presentation ingratiation.

### Mediating Effects of Workplace Anxiety

Workplace anxiety refers to the tension and stress that employees experience about a task that needs to be completed in the workplace (Eysenck et al., 2007; Muschalla and Linden, 2012; McCarthy et al., 2016). This type of anxiety occurs in the workplace (Zeidner and Matthews, 2005) and is affected by the organizational environment (Motowidlo et al., 1986). Workaholic leaders are likely to lead their subordinates to experience workplace anxiety. On the one hand, employees observe a leader’s behavior and obtain and interpret various signals transmitted by them. On the other hand, workaholic leadership often involves consciously lengthening working hours or using private time to do work, showing an evident dedication to work. Even if the leadership does not require employees to do the same, employees may receive the following signal: the leader encourages them to put as much time and energy into work as they do. If they do not, their careers are likely to suffer. Consequently, leaders are more likely to cause workplace anxiety, especially when employees have inadequate resources to cope with the job’s demands. This behavior inevitably diminishes their positive emotions at work (Van der Doef and Maes, 1999). Comparatively, because workaholic leaders spend so much time and energy on their work, they often have less energy to deal with their relationships with subordinates (Ng et al., 2007). Moreover, when facing pressure and frustration, workaholic leaders tend to direct their negative emotions toward subordinates or colleagues (Shimazu et al., 2010), leading to workplace aggression (Balducci et al., 2012). This atmosphere will lead to the continuous loss of employees’ physical and mental resources, resulting in tension and pressure, and finally, workplace anxiety. In addition, some studies confirmed that strong workaholic leaders would reduce their subordinates’ psychological detachment (Li et al., 2021), which ultimately may cause them workplace anxiety (Sonnentag et al., 2008).

According to the COR theory, employees with workaholic leaders may lose their physical and mental resources due to overwork and unsatisfactory relationship needs, leading to workplace anxiety and job burnout over time (Halbesleben, 2006) and fatigue (She et al., 2019). Hobfoll et al. (2018) indicates that resources loss exerts a more significant impact on individuals. In this case, individuals are more inclined to take defensive measures to avoid further loss of resources. On the one hand, employees may pretend to be enthusiastic about working because of the need for salary increases and promotions. On the other hand, self-presentation is an excellent way to avoid dissatisfaction or criticism of the leader and discourages the leader from assigning more work to subordinates. In summary, when employees are chronically anxious due to workaholic leadership, they tend to resort to defensive measures, such as self-presentation, to relieve emotional pressure and reduce the continuous loss of physical and mental resources. Accordingly, we propose the following hypothesis:

**H2:** Employee workplace anxiety mediates the relationship between workaholic leadership and self-presentation-type ingratiation. High workaholic leadership strengthens employee workplace anxiety, thereby encouraging employees to exhibit greater self-presentation.

### Moderating Effects of Segmentation Supplies

According to the COR theory, individuals will avoid the continuous loss of resources and try to obtain and recover depleted physical and mental resources (Hobfoll et al., 2018). For instance, the physical and mental resources that employees lose at work can be partially restored or enhanced through personal or family life outside of work. Thus, employees must have a clear boundary between work and family/leisure (Clark, 2000). When this boundary is blurred, work tasks may intrude on employees’ leisure time and hinder the recovery of their physical and mental resources, finally leading to emotional exhaustion (Ma et al., 2014). If employees can get enough rest and enjoy their leisure time after work, they can eliminate the physical and mental fatigue caused by work, thus improving their life satisfaction. Alternatively, they may face adverse physical and psychological effects (Bakker et al., 2013). Therefore, our study introduced segmentation supplies to represent the degree of support from the organization for employees to separate work from non-work areas (Kreiner, 2006). Previous studies found that high segmentation supplies helps reduce work-family conflict (Chen et al., 2009; Yun et al., 2012) and improve work-family enrichment (Kubicek and Tement, 2016). To some extent, segmentation supplies is essentially a resource provided by the organization for employees to support them not to work in their private time (Wu et al., 2018), which helps prevent their resources from being exhausted. According to the above discussion, when the organization supports employees’ need not to be disturbed by work in their private time (high segmentation supplies), their workplace anxiety will be interspersed with relaxing leisure time, and thus the physical and mental resources will be supplemented in the leisure time. The source of workplace anxiety will eventually decline, and employees may not need to self-present themselves frequently when they return to work. Conversely, low segmentation supplies means that employees may be working even during their private time. In such cases, as the anxiety caused by work will continue into the leisure time, employees will be more inclined to self-present themselves after sustained resource loss. Therefore, we propose that segmentation supplies negatively moderates the relationship between workplace anxiety and self-presentation.

**H3:** Segmentation supplies negatively moderates the relationship between workplace anxiety and self-presentation such that this relationship is stronger among employees with a lower level of segmentation supplies.

Our study also proposes that employees may suffer from continuous loss of physical and mental resources due to various demands from workaholic leaders (such as high-intensity work tasks and high-performance work goals), thus causing workplace anxiety. It could be explained by COR theory, as individuals strive to preserve their resources (e.g., energy resources), the impact of the loss of resources are more significant and urgent than resources acquisition (Hobfoll et al., 2018). Therefore, when resources loss occurs, individuals will exhibit tension and stress in response. They are more inclined to take defensive measures to avoid further loss of resources (Halbesleben, 2006; Hobfoll et al., 2018). At this point, when segmentation supplies remains low, no precise regulation prevents organizations from pressuring employees’ leisure time. Based on workplace anxiety, further physical and mental resources may be lost, which would force employees to ingratiate their superiors by self-presenting, as they may be too tired to focus on their task due to continuous loss of energetic resources in leisure time. On the contrary, if segmentation supplies remains high, resources loss may not be so serious, and employees’ workplace anxiety will be declined during leisure time, which will also lead to the reduction of self-presentation as the threat of resources loss was also reduced. That is, they may not self-present themselves frequently in front of their superiors. However, they are still under pressure from their workaholic leaders. Their loss of physical and mental resources could partly be restored or enhanced during leisure time, which means they have relatively adequate physical and mental resources to conduct their work with less dependence on self-present strategies. Therefore, we propose the following hypothesis:

**H4:** Segmentation supplies moderates the negative indirect effect of workaholic leadership on employee self-presentation via workplace anxiety, such that the negatively indirect effect is stronger when segmentation supplies is low rather that high.

**FIGURE 1:**
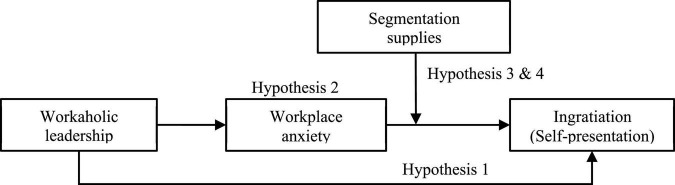
Theoretical model.

Figure 1 displays the theoretical model for this study.

## Materials and Methods

### Participants and Procedure

Our study participants were employees of five enterprises located in three provinces (Beijing, Guangdong, and Fujian) in China. Questionnaires were used to collect data. The questionnaires were collected at three time points to avoid common method bias (Podsakoff et al., 2003). With the support of internal contacts, employees were required to complete a paper or an online questionnaire. We promised that the participation was confidential and voluntary. Employees were required to complete the last four mobile phone numbers as three-time matching clues. In the first questionnaire (Time 1), participants were required to complete demographic information and workaholic leadership questions; 368 questionnaires were collected. After 4 weeks, the second questionnaire (Time 2) collected a total of 322 valid responses. Employees were asked to report workplace anxiety and segmentation supplies. Another 4 weeks later (Time 3), questionnaire including the dependent variable were distributed to employees. Finally, we received 256 employees’ surveys. The effective response rate of the questionnaire was 69.6%. Most employees were male (52.3%), and most participants were university or college graduates (57.8%). Most of the employees were under 40 years old (71.5%) and worked in their company within the last 10 years (58.6%). Regarding job positions, staff accounted for 45.3%, grassroots managers accounted for 27.3%, and middle managers accounted for 25.8% of the total sample. To test the common method bias, we adopted suggestions from Tang and Wen (2020) and validated them with Harman’s single-factor test. According to the result, the load of the first factor was 30.2% (<50%), indicating that only a small degree of common method bias.

### Measurement of Study Variables

All scales measured in the survey were well established by previous studies. According to Brislin (1986) suggestion, translation and back-translation procedures were used to ensure accuracy. In addition, five employees who were excluded from the survey were invited to check the comprehensibility and applicability.

#### Workaholic Leadership

The 10-item scale developed by Schaufeli et al. (2009) was completed by employees to assess their superiors’ workaholism. An example item included “He/she seems to be in a hurry and racing against the clock.” Responses were given on a five-point Likert scale, with options ranging from “Strongly disagree” to “Strongly agree” (Cronbach’s α = 0.87). Subordinates responded to questions about this variable because some workaholic leaders may not consciously be aware of their workaholism, a prominent trait.

#### Employee Self-Presentation

We used the 4-item scale developed by Bolino (1999) to measure the self-presentation dimension of employee ingratiation. An example item stated, “Sometimes I like to appear busy, even when work isn’t urgent, in case people think I’m slacking.” Chinese scholars have translated the scale with good reliability (Cui and Qu, 2014). Responses were recorded on a five-point Likert scale (Cronbach’s α = 0.89). Response options range from “Strongly disagree” to “Strongly agree.”

#### Workplace Anxiety

The 8-item Workplace Anxiety Scale developed by McCarthy et al. (2016) measured employees’ workplace anxiety. An example item stated. “I worry about whether others consider me to be a good employee for the job.” Responses are rated with a five-point Likert scale (Cronbach’s α = 0.93). The options ranged from “Strongly disagree” to “Strongly agree.”

#### Segmentation Supplies

Employees completed the 4-item scale developed by Kreiner (2006) measuring segmentation supplies. An example item was, “At my workplace, people are able to prevent work issues from creeping into their home life.” Responses were given on a five-point Likert scale (Cronbach’s α = 0.76). The options ranged from “Extremely disagree” to “Extremely agree.”

#### Control Variables

In this study, employees’ gender, age, education level, work experience, and position were the control variables. Self-presentation is an effective form of ingratiation. Liu et al. (2015) suggested that employees’ gender is associated with ingratiation behavior: men are more likely to ingratiate their superiors. Therefore, sex was used as the control variable. In addition, age may be an important variable that affects self-presentation because younger employees are less likely to ingratiate than older workers (Dubrin, 1991). Moreover, previous studies have suggested that ingratiation is more common in higher position levels (Allen et al., 1979). Thus, position level was regarded as a control variable.

### Statistical Analyses

We conducted confirmatory factor analysis with Amos software to determine the measurement model. After standardizing the sample data, we employed hierarchical regression analysis for subsequent hypothesis testing. We tested the mediating effect of workplace anxiety and the moderating effect of segmentation supplies with SPSS software. Finally, according to Edwards and Lambert (2007) suggestions, bootstrapping with 5,000 replications via PROCESS modeling of SPSS software was used to test the moderated mediating effects.

## Results

### Descriptive Statistics and Correlations

Table 1 shows the means, standard deviations, and correlation coefficients of the variables. A significant positive correlation was found between workaholic leadership and workplace anxiety (*r* = 0.39, *p* < 0.01) and employee self-presentation (*r* = 0.23, *p* < 0.01). Workplace anxiety was significantly and positively correlated with self-presentation (*r* = 0.36, *p* < 0.01). Employee self-presentation was negatively correlated with gender, which supports the findings presented by Liu et al. (2015).

**TABLE 1:** Means, standard deviations, and correlations between the study variables.

	Mean	*SD*	1	2	3	4	5	6	7	8	9
1. Gender	0.48	0.50									
2. Age	2.99	0.94	−0.26**								
3. Position	1.84	0.87	−0.31**	0.55**							
4. Education	2.40	0.62	–0.02	–0.07	0.05						
5. Working experience	2.48	1.48	−0.27**	0.84**	0.64**	–0.08					
6. Workaholic leadership	3.64	0.76	0.07	–0.08	–0.04	–0.04	–0.02	(0.87)			
7. Workplace anxiety	3.32	0.96	–0.12	–0.04	–0.04	–0.03	–0.06	0.39**	(0.93)		
8. Employee self-presentation	3.03	0.84	−0.21**	0.01	0.07	0.10	0.02	0.23**	0.36**	(0.89)	
9. Segmentation supplies	3.09	0.87	−0.16**	0.13*	0.06	–0.05	0.14*	0.30**	0.43**	0.04	(0.76)

Internal consistency reliabilities are in parentheses. N = 256. **p < 0.01, *p < 0.05.

### Measurement Model

Table 2 shows the confirmatory factor analysis results for the measurement model. According to the results, the *t*-value of the factor loading for all questions reached significance (*p* < 0.001). The composite reliability of the four variables was higher than 0.70 (workaholic leadership: CR = 0.91; self-presentation: CR = 0.73; workplace anxiety: CR = 0.93; segmentation supplies: CR = 0.77), all variables had good internal consistency. Regarding discriminant validity, Model 1 (four-factor) exhibited a better comparative fit index, incremental fit index, standardized root mean square residual, and root mean square error of approximation results compared to the other four models. This finding indicates that the overall fit of Model 1 (four-factor) was the best, and the questionnaire had discriminant validity and could be used for subsequent analysis (Anderson and Gerbing, 1988).

**TABLE 2:** Results of confirmatory factor analysis of the study variables.

Model	X^2^	*df*	X^2^/*df*	CFI	IFI	SRMR	RMSEA
1. Four-factor: Four factor separated	683.84	291	2.35	0.88	0.89	0.07	0.07
2. Three-factor: Workplace anxiety and self-presentation combined	1037.88	296	3.51	0.78	0.78	0.08	0.10
3. Three-factor: Workplace anxiety and segmentation supplies combined	1043.01	296	3.52	0.78	0.78	0.08	0.10
4. Two-factor: Workplace anxiety, segmentation supplies, and self-presentation combined	1239.80	298	4.16	0.72	0.72	0.10	0.11
5. One-factor: All four variables combined as one factor	1824.24	299	6.10	0.54	0.55	0.14	0.14

*N, 256. CFI, comparative fit index; IFI, incremental fit index; SRMR, standard root mean square residual; RMSEA, root mean square error of approximation.*

### Validation of the Theoretical Model and Study Hypotheses

Table 3 presents the main and mediating effects of the theoretical model. After controlling for gender and other variables, the results of Models 3 and 4 showed a significant positive correlation between workaholic leadership and employee self-presentation (*r* = 0.25, *p* < 0.001), indicating that workaholic leaders positively affect employee self-presentation. Thus, Hypothesis 1 was verified.

**TABLE 3:** Hierarchical regression test of mediating and moderating effects.

Variables	Workplace anxiety		Employee self-presentation				
	Model 1	Model 2	Model 3	Model 4	Model 5	Model 6	Model 7
Gender	−0.16*	−0.18**	−0.22***	−0.23***	−0.18**	−0.20***	−0.19***
Age	0.01	0.10	–0.03	0.02	–0.01	0.002	–0.01
Position	–0.14	–0.03	0.03	0.04	0.05	0.04	0.05
Education	–0.04	–0.02	0.09	0.10	0.10	0.10	0.10
Working experience	–0.09	–0.16	–0.03	–0.07	–0.03	–0.002	–0.01
Workaholic leadership		0.40***		0.25***	0.14*	0.17**	0.18**
Workplace anxiety					0.29***	0.35***	0.33***
Segmentation supplies						−0.19**	−0.19**
Workplace anxiety × segmentation supplies							−0.22***
Adjust R square	0.03	0.18	0.06	0.12	0.18	0.21	0.27
R square changes		0.16		0.06	0.07	0.03	0.06
*F*	1.34	9.33***	2.90*	5.48***	7.93***	8.22***	10.00***

N = 256. ***p < 0.001, **p < 0.01, *p < 0.05.

To confirm the mediating mechanism of workplace anxiety between workaholic leadership and employee self-presentation, we tested Models 3–5. Model 5 showed that workplace anxiety had a significant positive effect on employee self-presentation (*r* = 0.29, *p* < 0.001). In comparison with Model 4, the results of Model 5 showed that the coefficients between workaholic leadership and employee self-presentation were still significant. However, the corresponding regression coefficient was reduced (*r* = 0.14, *p* < 0.05), indicating that workplace anxiety partially mediated workaholic leadership and employee self-presentation. Thus, Hypothesis 2 was verified.

**FIGURE 2:**
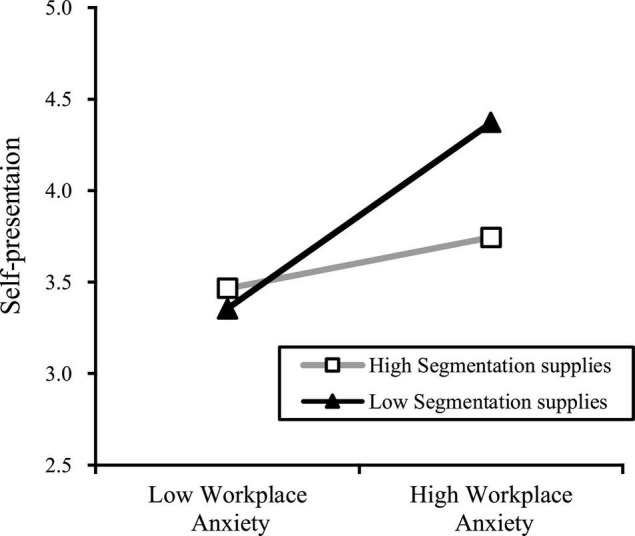
Moderating effect of Segmentation supplies between workplace anxiety and self-presentation.

The results of Models 6 and 7 showed that the interaction between workplace anxiety and segmentation supplies had a significant negative effect on employee self-presentation (*r* = −0.22, *p* < 0.001). This finding indicates that segmentation supplies negatively moderates the relationship between workplace anxiety and self-presentation. As Figure 2 shows, when segmentation supplies remains low, employees cannot receive support from their organization and tend to ingratiate their superiors by self-presenting themselves. However, if the segmentation supplies remains high, this motivation may no longer be obvious. Thus, Hypothesis 3 was verified.

Finally, regarding moderated mediating effects, Table 4 shows that the moderated mediating effect index was -0.11 with a 95% confidence interval between -0.18 and -0.06, excluding 0; that is, the moderated mediating effect was significant (Preacher and Hayes, 2004). Specifically, when segmentation supplies was low (-SD), the indirect effect of the model was significant, with a 95% confidence interval excluding 0, indicating that workaholic leadership leads to employee workplace anxiety, which would turn into employee self-presentation in this situation. When the segmentation supplies remains high (+ SD), the indirect effect of the model becomes insignificant (95% confidence interval includes 0), indicating that the whole mediation mechanism no longer exists. The difference in indirect effects between low and high segmentation supplies is significant. Thus, Hypothesis 4 was verified.

**TABLE 4:** Results of moderated mediating effects.

	Indirect effect	SE	95% Confidence intervals
High segmentation supplies (+ *SD*)	0.05	0.04	(−0.04, 0.14)
Low segmentation supplies (-*SD*)	0.25***	0.05	(0.15, 0.36)
Difference	−0.20***	0.05	(−0.31, −0.10)

N = 256. ***p < 0.001.

## Discussion

### Theoretical Contributions

This study makes several theoretical contributions to the literature. First, we found that high workaholic leadership exerts pressure on employees and creates anxiety. This process can be explained from the perspective of similar attraction and social generalization theories. To illustrate, employees will observe and mimic their superiors (Bandura, 1977); they may worry about being criticized by superiors for not working hard, which would gradually lead to workplace anxiety. This pattern is somewhat consistent with Fassel (1990), who suggested that workaholism harms the individual’s mental health of the individual. As what Andreassen (2013) propose, our study statistically confirms that workaholism can negatively influence surrounding people, as workaholic leaders tend to lead to workplace anxiety among employees.

Second, we found that employees may ingratiate their workaholic leaders by self-presenting to avoid being labeled as “slacking,” which means they prefer to appear busy, although they are not engaged. In addition, based on the COR theory, workplace anxiety is a mediating variable between workaholic leadership and employee self-presentation. Due to the motivation to avoid the continuous loss of physical and mental resources, self-presentation is an appropriate way to reduce workplace anxiety and win the approval of workaholic leaders. Previous studies have primarily focused on the effect of workaholic leadership on employee performance (She et al., 2020), satisfaction (Burke and Koksal, 2002), informal learning (Li and She, 2020), and creativity (Li et al., 2021). However, no studies have examined the effect of workaholic leadership on employee ingratiation (especially self-presentation). Our study fills academic gaps and provides a clear explanation of the mechanism by which employees deal with leader workaholism.

Third, workaholic leaders are likely to require their subordinates to continue to work in their leisure time (Friedman and Lobel, 2003), resulting in a continuous loss of employees’ physical and mental resources. Therefore, as a measured variable of the work-family boundary, the concept of segmentation supplies becomes a critical situational factor and leads to different strategies adopted by subordinates. Our study found that segmentation supplies moderated the entire mediating mechanism. Employees may be anxious about workaholic leaders interfering with their leisure time (Li et al., 2021). Therefore, high segmentation supplies means that employees could maintain a boundary between work and family life to recover their physical and mental resources. Furthermore, as there is no urgency for conserving resources, it is unnecessary to pretend to work hard. In contrast, low segmentation supplies allow work to interfere with employees’ private lives, strengthening the motivation to conserve resources. Employees may ingratiate their workaholic leaders through self-presentation to prevent the continued loss of these resources. They would do this by illustrating that they have done much work so that their leaders may not assign them a new task.

Finally, we used the COR theory to explain the mediating effects of employees’ workplace anxiety. Our study extends the COR theory to explain how external factors (such as workaholic leadership) can cause workplace anxiety and further expand to individual behavioral performance, such as self-presentation. This study also confirms that employee ingratiation (such as self-presentation) is the result of both external (such as workaholic leadership) and internal (such as workplace anxiety) factors. This finding enriches the research depth of workaholic leadership as well as employee ingratiation.

### Implications for Management

Our study has several practical implications. First and foremost, workaholic leadership exerts positive effects, such as encouraging employees to spend more time and energy on their work, which improves organizational performance (Li et al., 2018). However, it should also be clear that workaholic leadership can lead to a series of problems. As revealed in this study, workaholic leaders may not only cause employee workplace anxiety, but their behavior may also promote ingratiation (especially self-presentation), particularly when such workplace anxiety cannot be alleviated to some extent by leisure time. Even though employees spend a lot of time on work (pretending to work hard), it is difficult to guarantee efficiency and work quality, damaging organizational interests. Therefore, both organizations and leaders should not be obsessed with or exaggerate workaholic leadership; it is necessary to realize the potential loss to individuals and organizations. Thus, management should control working hours and output at a relatively reasonable and efficient level to avoid further diminishing the marginal benefit of working hours. Our study helps to understand the various circumstances and influences of workaholic leadership via the perspective of COR theory.

In addition, more efforts should be made to balance employees’ work and leisure lives to weaken the possible damage caused by workaholic leaders. Due to the limitations of time and energy and family and social responsibilities of employees, it is essential to create a proper balance between work and life. For example, forcing subordinates to work overtime, weekends, or holidays, may cause physical and mental stress, resulting in anxiety, work-family conflict (Hu and He, 2018), and marital estrangement (Yaniv, 2011). Therefore, organizations should respect employees’ personal lives and family responsibilities, reduce unnecessarily long working hours, and establish a boundary between work and family (Bloom et al., 2011). This policy would ultimately benefit the organization by improving employees’ work efficiency and effectiveness. Consequently, the new generation of employees can prioritize their private time and family responsibilities.

Finally, leaders must establish a correct view of work performance. On the one hand, leaders should not strive for high team performance by occupying subordinates’ leisure time without meaningful reasons. Instead, they should cooperate and encourage subordinates to find a more efficient way to complete more tasks and improve performance by improving their work efficiency instead of extending working hours. On the other hand, however, it is necessary to master scientific performance evaluation methods and be aware of possible deviations in performance evaluation. For example, the evaluation results should not only be obtained based on the usual behavior of subordinates but should consider the work results of employees. Suppose too much attention is paid to subordinates’ behaviors rather than results; it is easy for some subordinates to ingratiate leaders by self-presenting themselves to obtain better performance evaluation grades. In addition, leaders should pay attention to similarity errors in the performance evaluation process. They tend to give higher performance evaluations to those having characteristics similar to their own (Robin et al., 2014). Therefore, it is easy to give higher evaluations to those employees who exhibit workaholic characteristics, even though they may simply be pretending to work hard.

### Study Limitations and Future Research Suggestions

This study has the following limitations. First, due to the limitation of questionnaire collection, the sample sources of this questionnaire survey were extensive. Therefore, future research should be conducted in a specific industry to verify the influencing mechanism of workaholic leaders on employee behavior in different sectors. Second, this study is based on the COR theory, which explains the adverse effects of workaholic leadership on subordinates’ behavior. However, workaholic leaders’ enthusiasm for work may inspire subordinates in some situations (She et al., 2020). Therefore, further research should focus on the personality traits of subordinates that are suitable for workaholic leaders. Third, due to the differences in cultural backgrounds and institutional mechanisms, private, foreign, and state-owned enterprises may also have obvious differences in the management and work mode. Thus, further research should not ignore the influence of this category of work units. Fourth, some employees with a workaholic leader may refuse to self-present themselves, and this is likely to be condemned by the leader. Further study should focus on the supervisor ostracism via the perspective of workaholic leaders. Finally, ingratiating behavior includes self-presentation, enhancement, and opinion conformity (Jones, 1967). However, this study only discusses the self-presentation mechanism via COR theory. Thus, to enrich the research depth of the ingratiation theory, further research should focus on other types of ingratiation via diverse theoretical views (e.g., resources acquisition).

## Conclusion

This study investigated the effect of workaholic leadership on employee self-presentation and found that (1) a high workaholic leader will lead to employee self-presentation by causing subordinate workplace anxiety and (2) low segmentation supplies will strengthen the mediating process; in other words, it negatively moderates the whole mediating mechanism. These findings provide important insights into the underlying mechanism of workaholic leadership and employee behavior, which can be utilized to prevent adverse organizational outcomes and improve employee wellbeing, experience, and productivity.

## Data Availability Statement

The datasets of this study will be made available by the corresponding author/s to researchers upon reasonable request.

## Ethics Statement

This study was carried out in accordance with the recommendations of the Ethics Committee of Renmin University of China. The protocol was approved by the Ethics Committee of Renmin University of China. The patients/participants provided their written informed consent to participate in this study.

## Author Contributions

QZ substantially contributed to the conception, design of the work, data analysis, and preparation of the draft. XL critically reviewed and contributed important intellectual input. Both authors contributed to the article and approved the submitted version.

## Conflict of Interest

The authors declare that the research was conducted in the absence of any commercial or financial relationships that could be construed as a potential conflict of interest.

## Publisher’s Note

All claims expressed in this article are solely those of the authors and do not necessarily represent those of their affiliated organizations, or those of the publisher, the editors and the reviewers. Any product that may be evaluated in this article, or claim that may be made by its manufacturer, is not guaranteed or endorsed by the publisher.
